# Dope Fiends

**Published:** 1914-04

**Authors:** 


"Dope Fiends."—It makes me grit my teeth with vexation every time I see those objectionable words in print. "Dope" is an unpardonable vulgarism, and "fiend" is a gross misnomer. A "fiend" is a devil, a demon. Many of the victims of drugs are the gentlest women and, at least, inoffensive men, made victims in a good many cases by your too handy little hypodermic. These "fiends" are sick people and urgently need medical treatment. The disease is extensively prevalent, and as a result there are sanitariums all over the country for their treatment. But the class most affected cannot pay the sanitarium prices, and are thus driven to the quack doctor, whose medicine a la Wooley perpetuates the evil, or to patent medicines,—which do the same. They are objects of pity and not scorn.

SOMETHING SHOULD BE DONE by the State for these people, or by each city or each county. They are as truly sick people (and so are chronic alcoholics) as any other, and require hospital treatment. I urge upon my readers to put it up to the town or county authorities to provide wards for them; they will never cure themselves, and so long as they suffer they will get the drug, and some one must violate the law to supply it. "Dope fiends"—for shame.

